# A novel site of adult doublecortin expression: neuropeptide neurons within the suprachiasmatic nucleus circadian clock

**DOI:** 10.1186/1471-2202-9-2

**Published:** 2008-01-04

**Authors:** Darren Geoghegan, David A Carter

**Affiliations:** 1 School of Biosciences, Cardiff University, Cardiff, CF10 3US, UK

## Abstract

**Background:**

The mammalian suprachiasmatic nucleus (SCN) is composed of heterogeneous sub-groups of neurons that are organized into a neural system for the control of circadian physiology and behaviour. Molecular circadian 'clocks' are not an exclusive property of SCN neurons but the unique role of the SCN as a central integrative pacemaker is associated with specialized aspects of neuronal organization. Current studies are aimed at identifying the functional components of this hypothalamic integrative centre.

**Results:**

In the present study we have identified and characterized a quite novel aspect of SCN neurobiology, doublecortin (DCX) protein expression within a defined group of adult rat SCN neurons. Adult neuronal DCX expression is surprising because this microtubule-associated protein (MAP) is generally a developmentally restricted component of immature, migrating neurons. We have also demonstrated for the first time that the SCN as a whole exhibits low expression of the neuronal differentiation marker NeuN. However, DCX is co-localized with NeuN in the ventral SCN, and also with neuropeptides; DCX is extensively co-localized with GRP and partially co-localized with VIP.

**Conclusion:**

The highly selective expression of DCX in the adult SCN compared with other hypothalamic and thalamic nuclei shows that this MAP is somewhat uniquely required in certain SCN neurons, perhaps contributing to a specific functional property of the brain's circadian clock nucleus. DCX may maintain a capacity for dynamic cellular plasticity that subserves daily alterations in SCN neuronal signalling.

## Background

*Doublecortin *is a brain-specific gene, first identified in 1998, that is mutated in two human neurodevelopmental disorders: X-linked lissencephaly and double cortex syndrome [1]. 'Double cortex syndrome' provides the commonly used shorter name for *doublecortin*, '*DCX'*. *DCX *encodes a 40 kDa (UniProt predicted size) cytoplasmic protein (sometimes called doublin) that interacts with tubulin and is thereby a microtubule-associated protein (MAP) [2]. DCX regulates the assembly and stabilization of microtubule 'tracks', actions that appear to enhance molecular motor function [3,4]. DCX protein is now known to be part of a super-family of proteins that are related through a doublecortin homology domain [5].

DCX is present in immature (differentiating and migrating) neurons and is considered a marker for this stage of neurogenesis [see 6]. DCX regulates the migration of multiple classes of cortical neurons, facilitating migration via actions at the distal ends of neurites that promote neurite extension [3,7]. In the juvenile and adult brain, the expression of DCX is massively reduced compared with neonatal animals and has been shown to be restricted to a limited number of specialized neuronal cell groups, for example in the rostral migratory stream [8]. In the present study we have identified and characterized a group of adult DCX+ neurons that has not been recognized previously. These neurons are within a specific sub-division of the hypothalamic suprachiasmatic nucleus (SCN) which is the site of the 'master' circadian clock in the brain [9,10].

## Results

### DCX is expressed in the ventral suprachiasmatic nucleus

**Figure 1:**
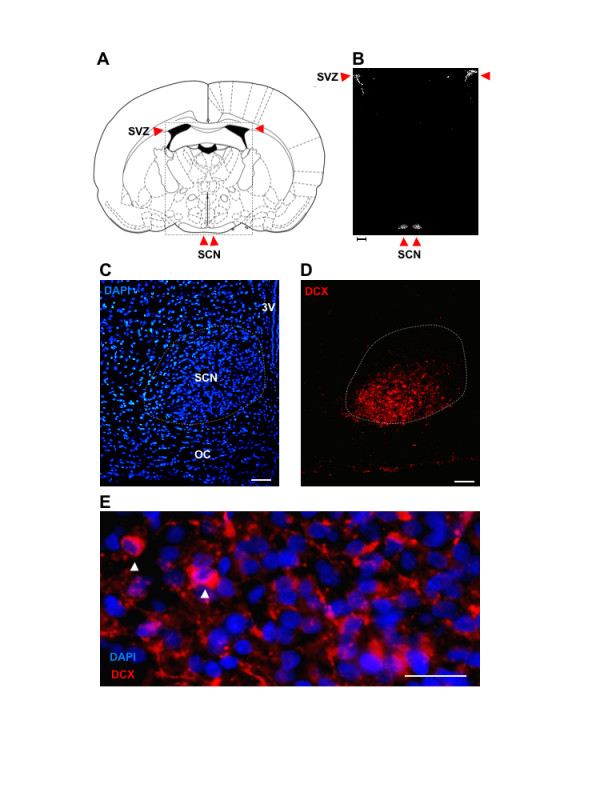
**Groups of DCX-positive cells in the rat brain are limited to a few sites including the ventral SCN**. **A**. Brain atlas image showing the SVZ and the SCN. **B**. Photomicrograph of DCX immunoreactivity within the region boxed in A. Note that groups of DCX+ cells are located only in the SVZ and SCN. Scale bar below image = 300 μm. **C & D**. Photomicrographs of DAPI-labelled and DCX-immunoreactive cells showing the location of DCX-immunoreactive cells within the ventral region of the SCN. Note the presence of some DCX-immmunoreactive cell bodies that are embedded in the optic chiasm. Scale bar = 50 μm. **E**. 20 μm. Abbreviations: 3V, third ventricle; OC, optic chiasm; SVZ, sub-ventricular zone.

Immunohistochemical analysis of rat brain sections (postnatal day [P] 25) using a rabbit anti-DCX antibody confirmed that expression of DCX is highly restricted at this age. One of the scarce groups of DCX+ neurons maintained at P25 [8] is located in the sub-ventricular zone (SVZ, Fig. 1B). In this study we have now made the novel observation that another cell group expressing abundant DCX immunoreactivity at this time is the suprachiasmatic nucleus (SCN) of the hypothalamus (Fig. 1B). The novelty of this finding is emphasized in Fig. 1B. by the scarcity of DCX+ cell groups at this age; note the absence of detected immunoreactivity across the entire intervening thalamic and hypothalamic regions between the SVZ and the SCN. Closer inspection of the SCN showed that DCX immunoreactivity is highly localized to the ventral SCN with some cell bodies embedded in the dorsal optic chiasm (Figs. 1C &1D). High magnification microscopy (Fig. 1E) revealed that DCX immunoreactivity is located both in the cytoplasm of SCN cell bodies and in processes that course through the ventral SCN.

### DCX expression is a constitutive property of the adult SCN

**Figure 2:**
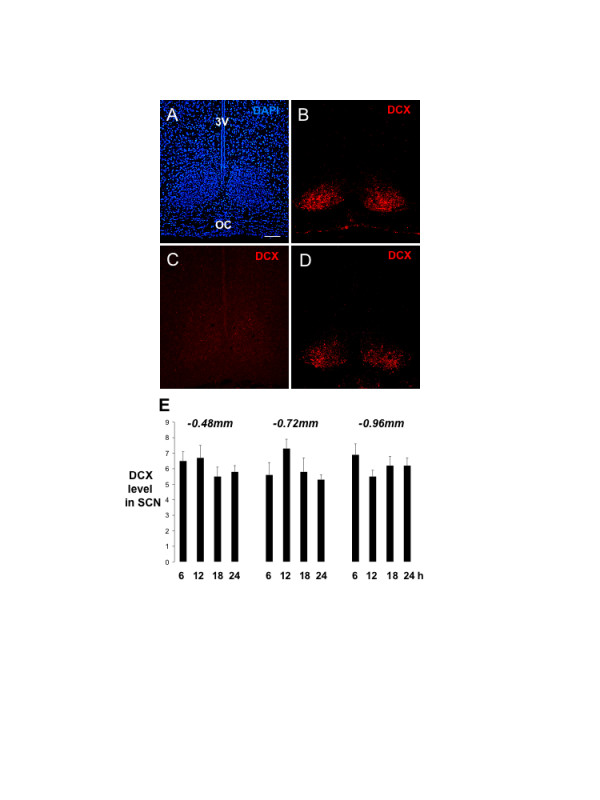
**Verification of SCN DCX immunoreactivity and comparative expression in the juvenile and adult rat**. **A-D**. Photomicrographs of DCX-immunoreactivity and DAPI staining in the ventral hypothalamus of the rat brain. Brain sections were from either P25 (A-C) or adult (6 month, D) rats. Brain sections were incubated with either control (A, B, D) or neutralized (C) anti-DCX antibody. **A-C**. Immuno-neutralization blocks detection of DCX in the SCN. Note that the camera exposure time of image C was longer relative to that of image B in order to reveal some background fluorescence. **D**. Note that DCX immunoreactivity in the adult is similarly distributed in the SCN, but at a lower relative level compared with the PN25 sample. Scale bar = 100 μm. Abbreviations: 3V, third ventricle; OC, optic chiasm. **E**. Absence of rhythmic DCX expression in the adult SCN. Histogram showing levels of DCX immunoreactivity at three rostro-caudal brain levels (-0.48, -0.72, -0.96 mm from bregma) measured at four time points. Levels (Leica Qwin Mean Grey level) are the mean ± S.E.M of six measurements (2 samples of SCN from each of 3 rats). Statistical analysis (ANOVA; see text) revealed no significant changes across time.

The identity of DCX immunoreactivity in this novel site was confirmed in two ways. First, it was shown that neutralization of the antibody with the peptide immunogen blocked detection of SCN reactivity (Fig. 2A–C). Second, using a different, previously characterized goat anti-DCX antibody [11,12], the same pattern of SCN DCX was observed (see Figs. 4G &4J). Furthermore, in order to confirm that the SCN DCX immunoreactivity was not a phenomenon restricted to one species/strain we demonstrated that DCX was similarly localized to the ventral SCN of mice (CD1 strain; not shown).

P25 rats were used in these initial studies because SCN innervations achieve adult-like levels at this juvenile stage of development [13]. In order to confirm that DCX expression is maintained in the adult SCN, we conducted further studies in adult rats, demonstrating that DCX expression is maintained in the adult rat SCN, at a slightly lower overall level compared with P25 brain (Fig. 2B vs. 2D).

Many proteins are expressed in a rhythmic (circadian) manner within SCN clock neurons [10]. We therefore examined DCX levels in adult rats during the light:dark cycle (Fig. 2E). Measurement of SCN DCX immunoreactivity at four time points, across three rostro-caudal locations, revealed no significant changes in DCX levels (Fig. 2E; ANOVA: 0.48 mm from bregma: F = 0.491, p = 0.870; 0.72 mm: F = 1.028, p = 0.527; 0.96 mm: F = 0.704, p = 0.736). Additionally, we did not observe any time-related changes in the location of DCX within the SCN. DCX protein expression is therefore a constitutive property of the adult SCN. During this analysis we also observed that DCX is highly restricted to the SCN with respect to adjacent rostral and caudal hypothalamic cell groups; it is not observed immediately rostrally in the ventromedial preoptic nucleus, nor immediately caudally in the retrochiasmatic area (not shown).

### Expression of the neuronal differentiation marker NeuN is low in the SCN

**Figure 3:**
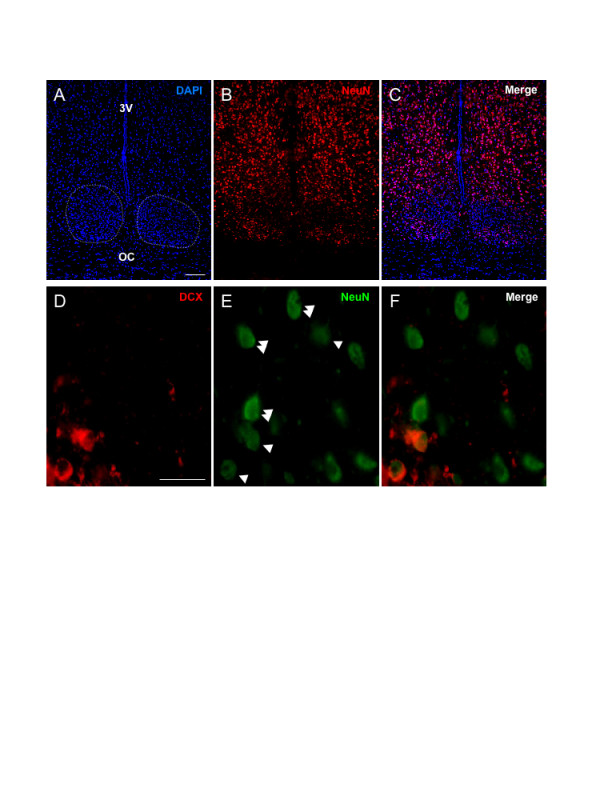
**The SCN is a region of low NeuN expression in the adult brain but SCN DCX is localized in NeuN+ neurons**. **A-C**: Photomicrographs of one field within the ventral hypothalamus of the adult rat brain showing NeuN in neurons and the absence of NeuN within OC glia. Note the low expression of NeuN in the SCN, particularly in the dorsal region. The boundaries of the SCN are marked with dashed lines. Scale bar = 100 μm. **D-F**: Photomicrographs of neurons within one field of the ventral SCN showing the localization of DCX within NeuN-positive neurons. Note the low relative level of NeuN in the DCX-positive, and some other cells (single arrowheads) compared with the higher level in the majority of DCX-negative cells (examples indicated with double arrowheads in E). Scale bar = 20 μm. Abbreviations: 3V, third ventricle; OC, optic chiasm.

The presence of DCX in the identified population of SCN cells suggested the possibility that these may be undifferentiated neuroblasts that, accordingly, do not express certain neuron-specific proteins. We therefore examined expression of NeuN, a marker of differentiation within most populations of CNS neurons [14] that is not expressed in the SVZ DCX+ cells (present study, not shown). Interestingly, we made the novel observation that the adult rat SCN expresses a low level of NeuN compared with the surrounding hypothalamic neurons (Fig. 3). At the same time, however, those cells in the SCN that did express detectable levels of NeuN were clustered in the ventral SCN, the region where DCX is also expressed (Fig. 3B &3C).

### DCX is expressed in NeuN+ neurons of the SCN

Dual immunohistochemical detection of DCX and NeuN (Figs. 3D–F) revealed that NeuN was indeed present in the majority of DCX-positive cells although rare NeuN-negative/DCX+ cells were observed. The majority of DCX+ cells in the adult SCN are therefore confirmed as neuronal. Many of the DCX+ SCN cells were observed to express a low relative level of NeuN compared to many surrounding DCX-negative cells (Fig. 2E).

### DCX is co-expressed with GRP and VIP in the SCN

**Figure 4:**
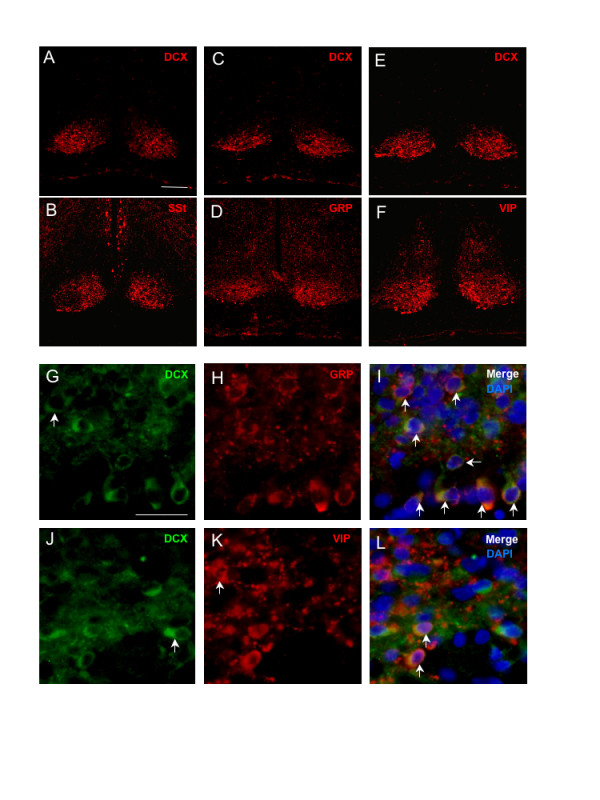
**DCX is expressed in GRP neurons and a sub-population of VIP neurons**. Photomicrographs of adult rat hypothalamus showing the comparative distribution of DCX and neuropeptide immunoreactivity. **A&B**. Adjacent sections showing DCX and somatostatin (SSt). **C&D**. Adjacent sections showing DCX and GRP. **E&F**. Adjacent sections showing DCX and VIP. Scale bar = 100 μm. **G-I**. Dual colour immunohistochemistry showing extensive co-localization of DCX and GRP. Co-localizing neurons are indicated by vertical arrows. An individual neuron with low but detectable GRP is indicated with a horizontal arrow. In G, a DCX+/GRP-negative neuron is indicated with a vertical arrow. **J-L**. Dual colour immunohistochemistry showing partial co-localization of DCX and VIP. Co-localizing neurons are indicated by vertical arrows in L. In J, a DCX+/VIP-negative neuron is indicated, and in K, a VIP+/DCX-negative neuron is indicated. Scale bar = 20 μm

The presence of DCX+ neuronal cell bodies in the ventral SCN suggested that these cells have identity to (one or more of) the neuropeptide-expressing cell groups that are distributed in the SCN. With reference to the mapping of these groups [9] we reasoned that the most likely co-localizing neuropeptide cell groups were somatostatin (SSt), vasoactive intestinal peptide (VIP) and gastrin-releasing peptide (GRP). In order to identify the cell-proup(s) in the SCN that express DCX we therefore compared DCX with these neuropeptides on adjacent brain sections (Figs. 4A–F). Notably, for each of the three SCN neuropeptides examined, immunoreactivity was considerably more dispersed across the ventral hypothalamus compared with DCX. This was particularly notable for SSt where strongly immunoreactive soma in the periventricular area did not express detectable DCX. Also, within the SCN, the distribution of DCX was quite unlike that of SSt which is heavily concentrated in the medioventral SCN. In contrast, although GRP+ and VIP+ fibres were again abundantly dispersed outside of the SCN, the intra-SCN distribution of these two neuropeptides closely resembled that of DCX, particularly in the case of GRP. Consequently, DCX co-localization studies were conducted with respect to GRP and VIP.

Dual immunohistochemical detection of DCX and GRP showed that these two proteins are widely co-expressed in the ventral SCN (Figs. 4G–I). Some DCX+/GRP-negative neurons were consistently observed (Fig. 4G) indicating that DCX is not confined to the GRP group (also see below). In contrast, it was not possible to discern DCX-negative/GRP+ neurons; low relative levels of GRP were present in some DCX+ neurons (Fig. 4H) but nevertheless this immunoreactivity was above background. In this respect it should be noted that GRP was not detectable in DCX+ neurons of the SVZ, and conversely, DCX was not detectable in GRP+ neurons of the lateral septal nuclear area (not shown).

DCX is also co-localized with VIP (Figs. 4J–4L), although to a much lesser extent than for GRP. Again, DCX+/VIP-negative cells were observed (Fig. 4J). Also, in the case of VIP, many VIP+ cells were DCX-negative (Fig. 4K). An abundance of VIP+ neurons embedded in the optic chiasm (Fig. 4F) compared with the relative absence of similarly localized DCX+ neurons in Fig. 4E. is illustrative of the relative separation of these two cell groups compared with the DCX and GRP groups.

## Discussion

### Suprachiasmatic nucleus – a novel site of DCX expression in the adult brain

DCX is a required component of migrating neurons that is accordingly highly expressed only during foetal and neonatal stages of brain development [2]. Western blot analysis of whole rodent brain shows that DCX protein levels are maximal at the time of birth (P0), markedly reduced by P10 and barely detectable in adult brain [see 15]. DCX expression is maintained in some adult neurons but these are scarce, specific exceptions that include a migratory stream of cells associated with the olfactory system [14]. Our finding that DCX is also expressed in neurons of the adult SCN is therefore a significant addition to our understanding of the adult roles of DCX. Furthermore, our findings add a potentially important new avenue of research into the molecular and functional organization of the SCN.

The mammalian SCN is a discrete and yet complex neuronal cell group that has been extensively characterized in recent years. This hypothalamic nucleus is the subject of intense study because it forms the brain's 'master' circadian clock, coordinating daily rhythms of central and peripheral physiology, rhythms that impact upon health and disease [10,16]. Molecular circadian clocks are not an exclusive property of SCN cells [16] but the intrinsic organization and extrinsic connections of the SCN provide it with a unique and central position in circadian control. The SCN contains many different neurotransmitters and neuropeptides [9,17], neurochemical components that subserve both inputs to the SCN [9,18] and also outputs that effect control over autonomic physiology [19]. Our finding that the SCN is, with respect to other hypothalamic and thalamic nuclei, an exclusive locus of adult DCX expression suggests that DCX is somewhat uniquely required in these SCN cells, and may therefore confer a specific property required for circadian function.

### Suprachiasmatic nucleus – a brain region of low NeuN expression

A second novel and interesting finding of the present study is that the SCN is a region of low NeuN expression. This finding would appear to be in accord with high DCX expression because down-regulation of DCX in differentiating neurons coincides with up-regulation of the mature neuronal marker protein NeuN [15]. However, we have found an apparently paradoxical inverse correlation in that NeuN is undetectable only in dorsal SCN neurons, whereas DCX is restricted to ventral SCN neurons, and is generally co-localized with NeuN. The functional significance of reduced NeuN expression in the SCN is unclear because although a limited number of other neurons including cerebellar Purkinje cells, and retinal phoreceptors do not express NeuN [14], the physiological role of this protein remains undefined. However, our finding defines an additional unusual property of SCN cells, in this case dorsal SCN neurons, that may relate to circadian function.

### DCX is expressed in GRP neurons and a sub-population of VIP neurons

DCX is expressed in the ventrolateral 'core' region of the SCN within particular neuropeptide-expressing cells. Our analysis has shown that DCX is located in both GRP+ and VIP+ neurons; all GRP+ neurons appear to be DCX+ whereas only a minority of VIP+ neurons co-express DCX. Previous studies have demonstrated a small population of VIP+/GRP+ neurons in the SCN [20]; additional reagents are required to determine whether DCX is present in such cells. The detection of a minority of DCX+/GRP-negative neurons indicates that the DCX+ population in the rat SCN is not identical to the GRP+ population. Nevertheless, the extensive co-localization of DCX and GRP is clearly indicative of a particular requirement for DCX in this population of neurons. Unlike SCN VIP which has been functionally characterized in mutant animal models [21], the role of the GRP in the SCN is less well defined. However, GRP, which is expressed in 14% of SCN neurons [9], appears to play a broadly similar role in linking SCN input to changes in clock neuronal activity [22-24]. An apparent redundancy between VIP and GRP function in the SCN may be reconciled by a specific property that is conferred on the GRP neurons by DCX.

### DCX function and plasticity-related molecules in the SCN

The functional role of DCX in the SCN is unknown. A specific action in circadian clock function can be inferred from the highly localized expression within this nucleus. Our observation that DCX levels do not change across the daily light/dark cycle cannot be taken as evidence that there is no rhythmic change in DCX activity because potential changes in DCX phosphorylation state mediated by JNK and/or cdk5 [7,25] may affect DCX function [7]. The possible involvement of JNK in modulating the activity of SCN DCX is intriguing because there is evidence that JNK regulates circadian rhythms [26].

In a previous study of adult neuronal DCX expression [8] it was suggested that the maintenance of adult expression is required for microtubule-related 'plasticity' in synaptic organization. In this respect, another plasticity-related molecule, the polysialated form of the neural cell adhesion molecule (PSA-NCAM), which is co-expressed with DCX during neuronal development [6], is also found in the adult SCN [27]. Therefore, DCX, together with other plasticity-related molecules, may mediate rhythmic changes in SCN synaptic organization that underlie day/night changes in electrical signalling [28]. Finally, our current findings may relate to the capability that exists for functional transplantation of the SCN [29]. The cellular requirements for this property have been considered before [30]; now it may be of value to investigate the DCX+ SCN sub-population that we have identified here.

## Conclusion

In this study we have identified two novel aspects of suprachiasmatic nucleus (SCN) organization: (i) adult expression of the immature neuronal marker protein, DCX, in a ventral SCN population of GRP/VIP neurons, and (ii) low SCN expression of the mature neuronal marker protein, NeuN. These findings are important for two reasons. Firstly, they are relevant to our understanding of the molecular mechanisms that control neuronal differentiation, identifying, for example, a model system that can be used to address the pathways that permit adult expression of DCX. Secondly, the restriction of these immature neuronal characteristics to particular regions/neurons of the SCN suggests a specific relevancy to the functional competence of these SCN components.

## Methods

### Experimental animals

Animal studies were conducted in accordance with both UK Home Office regulations, and local ethical review. Animals were maintained in a 14:10 light:dark cycle (lights on: 05.00 h). Male Sprague-Dawley rats were used (juvenile [P25], or adult, 3–6 month) apart from one experiment where adult CD1 mice (4 month) were used to confirm DCX expression in a second species. Each immunohistochemical analysis was conducted on a minimum of four brain sections sampled from each of two animals. Brains were sampled at 12.00 h apart from one experiment where an analysis of rhythmic expression was conducted. Rats were anaesthetized with sodium pentobarbitone (150 mg/kg, i.p.) and perfused via the ascending aorta with phosphate buffered saline followed by 4% paraformaldehyde in 0.1 M phosphate buffer (PFA). Tissues were then post-fixed in PFA for 90 min at 4°C, and cryoprotected in 20% sucrose in 0.1 M phosphate buffer at 4°C. Tissues were then frozen on dry-ice, and stored at -70°C prior to sectioning.

### Immunohistochemical analysis

Brains sections (10 μm) were cut using a Bright OTF cryostat (Bright Instrument Company Limited, Huntingdon, UK) and mounted on glass slides (SuperFrost Plus, VWR International, Poole, Dorset, UK). Sections were treated with 0.01 M citrate buffer, pH 6.0, at 60°C for 2 h. Proteins/peptides were detected either using either a standard immunohistochemical procedure [31] or alternatively using the tyramide signal amplification (TSA) procedure according to the manufacturer's protocol (PerkinElmer, Waltham, MA, USA). Dual detection of proteins was accomplished by using the TSA procedure, followed by the standard procedure for a second protein. Prior to the TSA procedure, sections were additionally treated with hydrogen peroxide, 1.5% in methanol, for 20 mins.

The following primary antisera were used: rabbit anti-doublecortin (rabbit), ab18723, abcam, Cambridge, UK; anti-doublecortin (goat), sc-8066, Santa Cruz Biotechnology, Santa Cruz, CA, USA; anti-GRP, ab22623, abcam; anti-NeuN, MAB377, Chemicon International, Temecula, CA, USA; anti-somatostatin, T4103, Peninsula; anti-VIP, T-4246, Peninsula. These were used in combination with the appropriate biotin/fluorophore-conjugated, secondary antisera: biotin-conjugated donkey anti-rabbit IgG, abcam; Alexa Fluor 488-conjugated donkey anti-goat IgG, Molecular Probes Inc, Eugene, OR, USA; Alexa Fluor 568-conjugated goat anti-mouse IgG, Molecular Probes; Cy3-conjugated sheep anti-rabbit IgG, Sigma. In control experiments, aliquots of the rabbit doublecortin antibody and the GRP antibody were incubated with an excess of the peptide immunogens (doublecortin: ab19804, abcam; GRP: G8022, Sigma, St. Louis, MO, USA; overnight, 4°C) prior to the TSA procedure. For both antibodies, this absorption/neutralization procedure blocked detection of the respective protein/peptide (doublecortin: see Fig. 2. GRP; not shown). Following final washing, sections were mounted under coverslips using Vectashield with DAPI (Vector Laboratories, Burlingame, CA, USA).

Brain sections were viewed using either an epifluorescence (Leica DM-LB, Leica Microsystems Imaging Solutions Ltd, Cambridge, UK) or laser confocal microscope (Leica TCS-SP2-AOBS). Images were captured using either a Leica DFC-300FX digital camera and Leica QWin software (V3) or Leica Confocal Software, and montaged in Photoshop (CS2, Adobe Systems Inc., San Jose, CA, USA). QWin software was also used to obtain measurements of neuronal fluorescence intensity: greyscale images (X200) were captured and Mean Grey levels were determined in a sample rectangle (30–40 k pixels). A similar sample was measured in the adjacent optic chiasm region to serve as a (subtracted) control for background. Two samples of SCN fluorescence, at three rostro-caudal levels were obtained from 12 rat brains (3 rats at each of 4 time points: see Results). Rat brain map images were downloaded from the Rat Brain Atlas [32] and modified in Photoshop.

## Authors' contributions

DG carried out some of the immunohistochemical analysis, and contributed to the intellectual progression of the study. DAC carried out animal sampling, immunohistochemical analysis and drafted the manuscript. Both authors read and approved the final manuscript.
